# The roles of innate and adaptive immunity in inactivated viral vaccination‐mediated protection against COVID‐19

**DOI:** 10.1002/ctm2.1530

**Published:** 2024-01-15

**Authors:** Shanhe Yu, Shijun Chen, Jiang Zhu, Jieming Qu

**Affiliations:** ^1^ Shanghai Institute of Hematology, State Key Laboratory of Medical Genomics National Research Center for Translational Medicine at Shanghai Collaborative Innovation Center of Hematology Ruijin Hospital affiliated to Shanghai Jiao‐Tong University School of Medicine Shanghai China; ^2^ Department of Pulmonary and Critical Care Medicine Ruijin Hospital Institute of Respiratory Diseases School of Medicine Shanghai Jiao‐Tong University Shanghai China; ^3^ Key Laboratory of Emergency Prevention, Diagnosis and Treatment of Respiratory Infectious Diseases Shanghai China

**Keywords:** COVID‐19, inactivated viral vaccination, innate and adaptive immunity, trained immunity

1

The COVID‐19 pandemic, fueled by the emergence of SARS‐CoV‐2 Omicron variants, has raised concerns surrounding immune evasion and the effectiveness of existing vaccination strategies.^1^ The initial emergence of the Omicron variant BA.1 in November 2021, characterised by significant mutations in the spike glycoprotein, has led to partial evasion of previously acquired immunity from the original SARS‐CoV‐2 strain. Subsequently, a succession of Omicron sublineages, including BA.2, BA.5, BQ.1 and EG.5 have come to the forefront on a global scale. COVID‐19 manifests a spectrum of symptoms from mild to severe, including cough, fever, dyspnoea and sometimes respiratory failure, alongside a substantial incidence of asymptomatic cases. To combat this, a diverse array of SARS‐CoV‐2 vaccines, including mRNA, adenovirus‐based and inactivated vaccines, have been developed. Accumulating evidence suggests that administering a third dose of either an mRNA or inactivated vaccine may confer protection by reducing the severity of the symptoms and mortality.^2^, ^3^ However, the intricate immunological mechanisms behind these observations mandate comprehensive investigation.

Recent studies, including our own work published in Cell,^4^ have emphasised the crucial role of adaptive immunity in COVID‐19 vaccination. Neutralising antibody levels are a strong indicator of immune defense against symptomatic SARS‐CoV‐2 infection.^5^ Inactivated vaccines, although eliciting lower neutralising antibody levels than mRNA vaccines, provide comparable protection with a three‐dose regimen.^2^, ^3^ It is reported that the protection of T‐cell response induced by inactivated vaccines is characterised by a robust CD4 response, though accompanied by a weaker CD8 response.^6^ In our study, Omicron‐infected individuals who received a third inactivated vaccine dose showed significant expansion in three effector memory CD4^+^ T‐cell subsets: CD161^hi^ effector memory, CD27^int^ effector memory and Th1‐like effector memory cells. This suggests that vaccination fuels the formation of long‐term CD4^+^ T‐cell memory pools, capable of responding to subsequent SARS‐CoV‐2 infections, including the Omicron variant. Importantly, post vaccination, especially after the third dose, CD4^+^ T cells tend to skew toward a Th1‐type response characterised by IFN‐γ secretion, rather than Th2, Th17 or TFH responses upon Omicron infection. This aligns with prior findings that both mRNA and inactivated vaccines induce a Th1‐polarised response, which is likely associated with asymptomatic infection. Our results also indicate that the frequency of regulatory T cells (Tregs) and their expression of TIM‐3 were increased post infection, but this effect is diminished in triple‐vaccinated individuals, suggesting that the vaccination might counteract the infection‐induced Treg expansion and activation, which could suppress CD4^+^ T‐cell activation and recall. Genes associated with Treg functional stability were upregulated, while Treg proliferation genes were downregulated in the three‐dose group. These findings support the notion that the enhanced number and pathogenic activation of Tregs upon Omicron infection, typically associated with delayed CD4^+^ T‐cell activation, can be effectively curtailed by a three‐dose vaccination regimen.

While extensive research has predominantly focused on the adaptive immune response, particularly its humoral components in COVID‐19 vaccination, the role of innate immunity has received comparatively less attention. Innate immune cells, including neutrophils, monocytes, macrophages, dendritic cells (DCs) and natural killer (NK) cells, play pivotal roles in the initial defense against viral infections, contributing to shaping the subsequent adaptive immune response. Studies report that two mRNA vaccine doses were able to strengthen the innate immune response, increasing the frequency of inflammatory intermediate monocytes and enhancing innate antiviral signatures.^7^ With three mRNA vaccine doses, NK cell maturation is promoted, underscoring the importance of this innate immunity response.^8^ In our study,^4^ we found that the Omicron‐infected individuals displayed reduced frequencies of HLA‐DR^int^ classical monocytes (HLA‐DR^int−^CM), HLA‐DR^hi^ classical monocytes (HLA‐DR^hi^‐CM) and non‐classical monocytes (NCM) compared to healthy controls. Notably, the frequencies of HLA‐DR^int−^CM and NCM were partially restored with a third vaccine dose. HLA‐DR^hi^‐CM and NCM frequencies correlated positively with viral replication inhibition, suggesting their role in antiviral defense. Additionally, Omicron infection decreased plasmacytoid dendritic cell (pDC) frequencies, which are partially reconstituted after the third dose. Moreover, there was a shift in the frequency of NK cell subsets, with a decrease in cytokine‐secreting CD56^hi^CD16^lo^ NK cells and an increase in CD56^int^CD16^hi^ cytotoxic NK cells following three vaccine doses in Omicron‐infected individuals. These results indicate that three doses of the inactivated vaccine promote the activation and maturation of monocytic, dendritic and NK cells. Notably, we observed negative correlations between the frequencies of HLA‐DR^hi^‐CM, NCM, pDC and the frequency of Tregs, hinting at an inverse relationship between monocytic/dendritic activation and pathogenic Treg expansion.

Many studies over the past century have demonstrated that specific vaccines, especially those utilising live attenuated microorganisms like the Bacillus Calmette–Guérin (BCG) vaccine, robustly activate myelopoiesis and boost innate immune cell functionality upon pathogen challenge, a phenomenon known as ‘trained immunity’.^9^ The mechanism of trained immunity in myeloid cells involves the remodeling of the epigenetic landscape, driven by key transcription factors such as C/EBPβ, PU.1 and interferon regulatory factors (IRFs), coupled with metabolic rewiring, which leads to enhanced killing capacity and production of cytokines and chemokines.^10^ While BCG‐trained innate immunity was considered as a potential way to prompt protection against SARS‐CoV‐2, it remained unclear whether the inactivated vaccine could induce trained immunity programmes against Omicron. Our study provides evidence that a substantial portion of genes activated by vaccination in healthy individuals undergo similar changes following Omicron infection. Interestingly, most of these shared genes, including critical regulators like *TREM1*, *C5AR1* and *FOSL1*, are most highly expressed in monocytes among the 26 immune cell types of peripheral blood mononuclear cells (PBMCs). This observation suggests that the essential monocytic‐driven pathways, such as *TREM1* upregulation upon Omicron infection, might be primed by prior vaccination‐induced monocytic training in healthy individuals. Furthermore, our research reveals that trained immunity, induced by booster vaccination, predominantly stimulates monocytic activation and differentiation, rather than the suppression of monocytes typically seen in SARS‐CoV‐2 infection.

In conclusion, the newly published work enriches our understanding of the immunomodulatory effects of inactivated virus vaccines. Our study systematically elucidates the impact of inactivated COVID‐19 vaccine inoculation on the innate and adaptive immune responses in Omicron‐infected subjects (Figure 1). Moreover, we uncover the intricate molecular mechanisms behind the potent antiviral effects induced by three booster doses through ‘trained immunity’. This pivotal discovery is expected to provide a scientific basis for future vaccine development and immunisation strategies.

**FIGURE 1:**
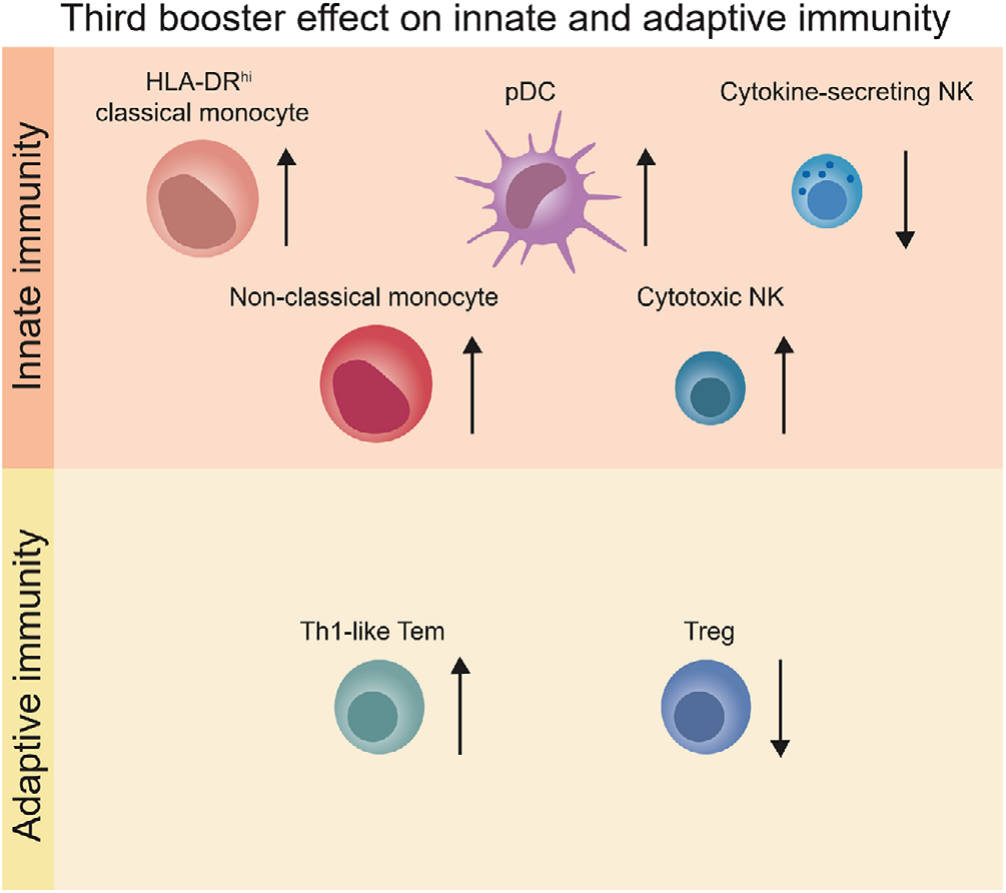
The role of innate and adaptive immunity in mediating protection against COVID‐19 through inactivated viral vaccines.

## AUTHOR CONTRIBUTIONS

S.Y wrote the manuscript. J.Z and J.Q provided important instructions and revised the manuscript. S.C critically read the manuscript.

## CONFLICT OF INTEREST STATEMENT

The authors declare they have no conflicts of interest.

## ETHICS STATEMENT

None
